# The E2F2 Transcription Factor Sustains Hepatic Glycerophospholipid Homeostasis in Mice

**DOI:** 10.1371/journal.pone.0112620

**Published:** 2014-11-14

**Authors:** Eduardo N. Maldonado, Igotz Delgado, Natalia E. Furland, Xabier Buqué, Ainhoa Iglesias, Marta I. Aveldaño, Ana Zubiaga, Olatz Fresnedo, Begoña Ochoa

**Affiliations:** 1 Department of Physiology, Faculty of Medicine and Dentistry, University of the Basque Country, Leioa, Spain; 2 Department of Genetics, Physical Anthropology and Animal Physiology, Faculty of Science and Technology, University of the Basque Country, Leioa, Spain; 3 Instituto de Investigaciones Bioquímicas de Bahía Blanca, Consejo Nacional de Investigaciones Científicas y Técnicas y Universidad Nacional del Sur, Bahía Blanca, Argentina; Institute of Hepatology - Birkbeck, University of London, United Kingdom

## Abstract

Increasing evidence links metabolic signals to cell proliferation, but the molecular wiring that connects the two core machineries remains largely unknown. E2Fs are master regulators of cellular proliferation. We have recently shown that E2F2 activity facilitates the completion of liver regeneration after partial hepatectomy (PH) by regulating the expression of genes required for S-phase entry. Our study also revealed that E2F2 determines the duration of hepatectomy-induced hepatic steatosis. A transcriptomic analysis of normal adult liver identified “lipid metabolism regulation” as a major E2F2 functional target, suggesting that E2F2 has a role in lipid homeostasis. Here we use wild-type (E2F2^+/+^) and E2F2 deficient (E2F2^−/−^) mice to investigate the *in vivo* role of E2F2 in the composition of liver lipids and fatty acids in two metabolically different contexts: quiescence and 48-h post-PH, when cellular proliferation and anabolic demands are maximal. We show that liver regeneration is accompanied by large triglyceride and protein increases without changes in total phospholipids both in E2F2^+/+^ and E2F2^−/−^ mice. Remarkably, we found that the phenotype of quiescent liver tissue from E2F2^−/−^ mice resembles the phenotype of proliferating E2F2^+/+^ liver tissue, characterized by a decreased phosphatidylcholine to phosphatidylethanolamine ratio and a reprogramming of genes involved in generation of choline and ethanolamine derivatives. The diversity of fatty acids in total lipid, triglycerides and phospholipids was essentially preserved on E2F2 loss both in proliferating and non-proliferating liver tissue, although notable exceptions in inflammation-related fatty acids of defined phospholipid classes were detected. Overall, our results indicate that E2F2 activity sustains the hepatic homeostasis of major membrane glycerolipid components while it is dispensable for storage glycerolipid balance.

## Introduction

The mammalian liver is a lipidostat that plays a central role in whole body lipid metabolism. Healthy livers regenerate efficiently after partial hepatectomy (PH). Successful regeneration requires replenishing all of the various epithelial and stromal cell types that compose the liver and a complex matrix remodeling to restore tissue homeostasis. Following resection of 70% of adult liver, 90-95% of the remaining hepatocytes leave their quiescent state and quasi-synchronously reenter the cell-cycle to begin regeneration [1]. Hepatocytes are the first cells reentering the cell-cycle, followed by biliary epithelial cells and stromal cells (Kupffer cells and stellate cells) 48 hours later, and sinusoidal endothelial cells, 96 hours later [2], [3]. Maximum DNA synthesis takes place within the initial 40-48 hours after PH in mice [3]–[5], which poses a dramatic demand of biomass formation to make daughter cells: fatty acids (FA), amino acids and other molecular building blocks. As blood glucose and hepatic glycogen levels decrease drastically a few hours after PH, peripheral lipid metabolism becomes essential for liver cells to fuel required ATP generation [6]–[8]. Likewise, the hepatic accumulation of lipid droplets (LDs) plays a key role for transiently storing lipids that are necessary for metabolic energy and membrane precursors [9].

Many cell-cycle regulators are known to contribute to liver regeneration [10]–[13]. We have recently demonstrated that E2F2 transcription factor is required for mature hepatocytes to exit quiescence and enter the cell-cycle after PH [5]. Disruption of the *E2F2* gene in hepatocytes led to a reduced rate of S-phase entry and to delayed liver regeneration, along with prolonged hepatectomy-induced steatosis. By contrast, other members of the E2F family (E2F1 and E2F4) are dispensable for this function [14], [15]. E2F2 is a member of a family of transcription factors (E2F1-8) that were originally described as regulators of genes that are critical for cell-cycle progression [16]. Several members of the family, including E2F2, display both activator and repressor transcriptional activities, depending on the cellular context. They function as negative regulators of transcription when bound to hypophosphorylated retinoblastoma in quiescence, or in association with other transcriptional regulators [17]; [18]. By contrast, they activate transcription when released from the repressor complexes after retinoblastoma is phosphorylated by cyclins and cyclin-dependent kinases in G_1_ [17], [18]. This duality of functions is also reflected in their functional role in cell-cycle control. For example, E2F2 contributes to promote cell division in mouse embryonic fibroblasts [19], hematopoyetic progenitor cells [20] and regenerating hepatocytes [5]. Conversely, this E2F is essential for the maintenance of quiescence in lymphoid and pancreatic cells, and its loss results in unscheduled entry in the cell-cycle [21]–[23].

Transcriptomic studies have revealed that E2F factors not only regulate the expression of genes involved in cell-cycle control. Genes involved in differentiation, apoptosis, autophagy or metabolism are also regulated by E2Fs [21], [24], [25]. In E2F2^-/-^ regenerating liver [5], aberrant expression of a number of genes involved in the metabolism of triacylglycerols (TAG) and phospholipids (PL) suggested that both lipid synthesis and metabolism might be differentially regulated in proliferating E2F2^-/-^ hepatocytes, and that E2F2-regulated transcripts in the quiescent tissue could impact lipid homeostasis during regeneration.

The role of E2F transcription factors in the regulation of bioenergetics and metabolism is being increasingly recognized [26]. However, no previous studies have addressed a mechanistic dependence of lipid metabolism on E2F activity. Our previous research using E2F2-deficient mice revealed that the E2F2 transcription factor is essential for the liver to develop defined phenotypic marks that appear during regeneration after PH, including the timely mobilization of LDs lipids and the regulated expression of a set of genes involved in lipid metabolism [5].

Lipid homeostasis is a dynamic phenomenon in which import, synthesis, traffic, degradation and exportation are tightly regulated in order to maintain a specific lipid composition optimal for their individual functions. In the current work, we have investigated whether E2F2 contributes to sustain liver PL and FA compositions *in vivo*. Using E2F2^+/+^ and E2F2^-/-^ mice, we have studied two metabolically distinctive contexts: (i) quiescent tissue, when E2F2 transcriptional activity is presumably repressed and metabolic requirements are basal, and (ii) proliferating 48-h post-PH tissue, when the high metabolic demand that follows resection of 70% of the liver challenges E2F2 activity [27] and the reprogramming of whole body lipid metabolism to support proliferation [6]–[8]. Our findings indicate that *E2F2* gene activity is necessary to sustain normal PL composition in the adult mouse liver, affecting particularly the phosphatidylcholine (PC) to phosphatidylethanolamine (PE) ratio and the content of defined FA in glycerolipid and sphingolipid classes.

## Material and Methods

### Animals and surgical procedure

**Figure 1:**
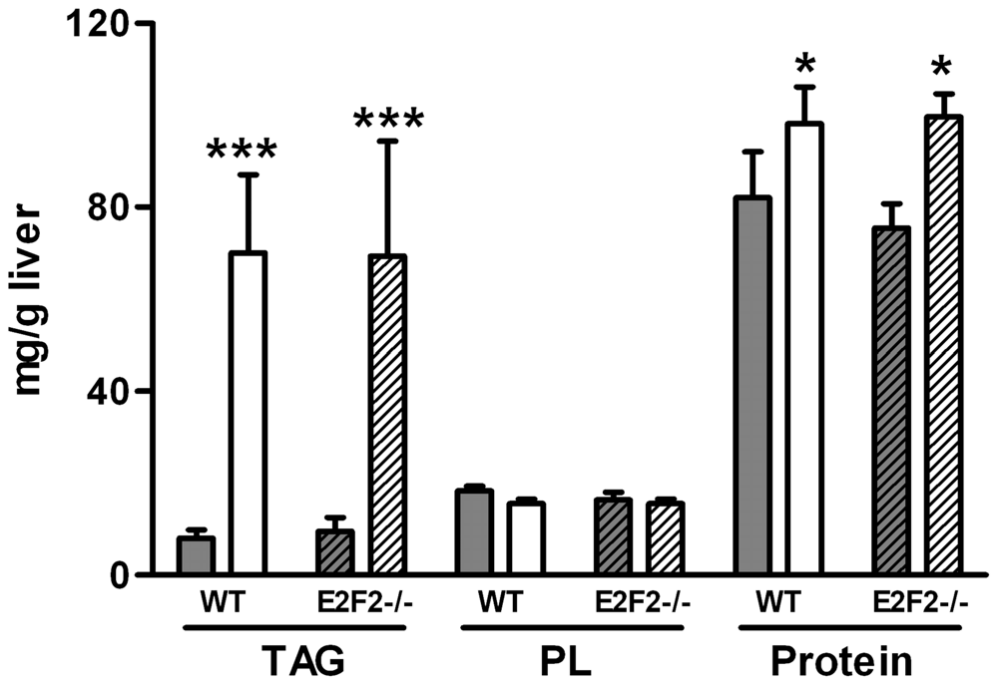
E2F2 gene deletion does not promote changes in total triacylglycerol, phospholipid and protein content in quiescent liver or at 48-h after 70% partial hepatectomy. Partial hepatectomy was performed on E2F2^+/+^ (wild-type, WT) and E2F2^-/-^ mice, and were sacrificed 48 hours later. Quiescent (0-h, solid bars) and regenerating (48-h, open bars) livers were harvested and homogenized and the triacylglycerol (TAG), phospholipid (PL) and protein content of homogenates were quantified as described in Materials and Methods. Data are shown as means ± SD and are representative of three independent experiments using cohorts of 6 WT and 6 E2F2^-/-^ mice each. Statistical differences between the regenerating and the quiescent tissue of a genotype are denoted by * *P*≤0.05, *** *P*≤0.001 (unpaired, 2-tailed Student's *t*-test).

**Figure 2:**
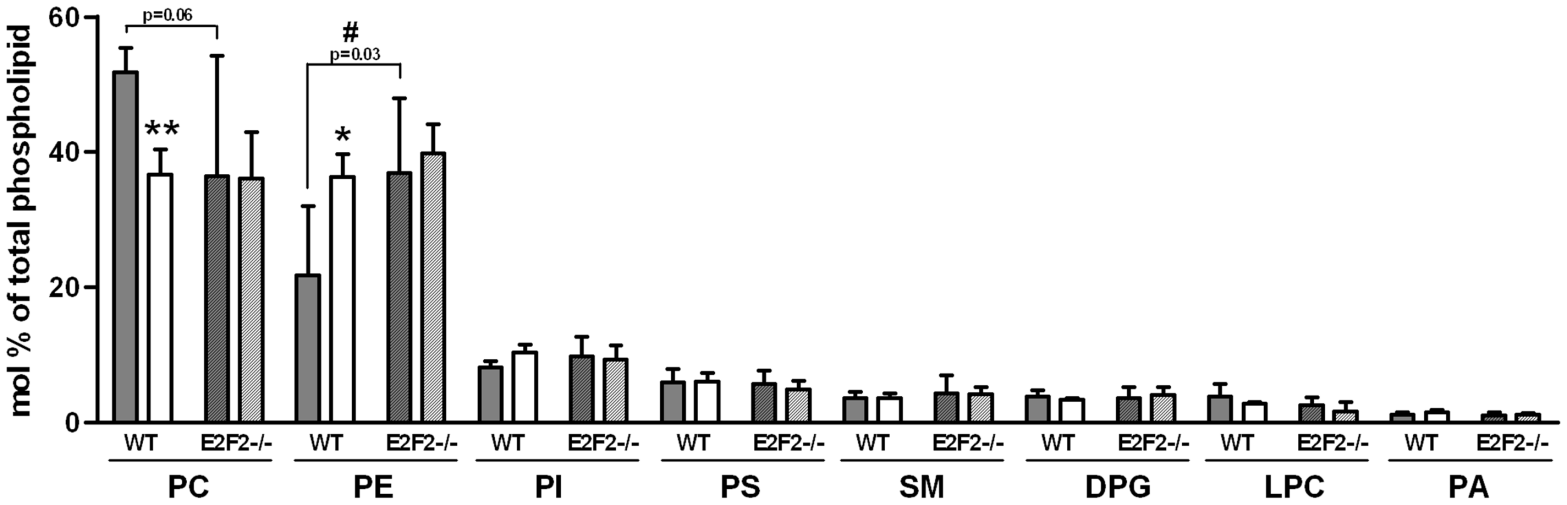
E2F2 gene deletion alters the phospholipid composition of quiescent liver. Partial hepatectomy was performed on E2F2^+/+^ (wild-type, WT) and E2F2^-/-^ mice, and were sacrificed 48 hours later. Quiescent (0-h, solid bars) and regenerating (48-h, open bars) livers were harvested and homogenized and lipids extracted from homogenates. The composition of the major phospholipid classes phosphatidylcholine (PC), phosphatidylethanolamine (PE), phosphatidylinositol (PI), phosphatidylserine (PS), sphingomyelin (SM), cardiolipin (diphosphatidylglycerol, DPG), lysophosphatidylcholine (LPC) and phosphatidic acid (PA) were determined by phosphorous analysis after their separation by thin-layer-chromatography. Results are shown as mol% of total phosphorous. Data and presented as means ± SD and are representative of three independent experiments using cohorts of 6 WT and 6 E2F2^-/-^ mice each. Statistical differences between the regenerating and the quiescent liver of a genotype are denoted by * *P*≤0.05, ** *P*≤0.01, and between the same tissue condition of different genotypes are denoted by # *P*≤0.05 (unpaired, 2-tailed Student's *t*-test).

**Figure 3:**
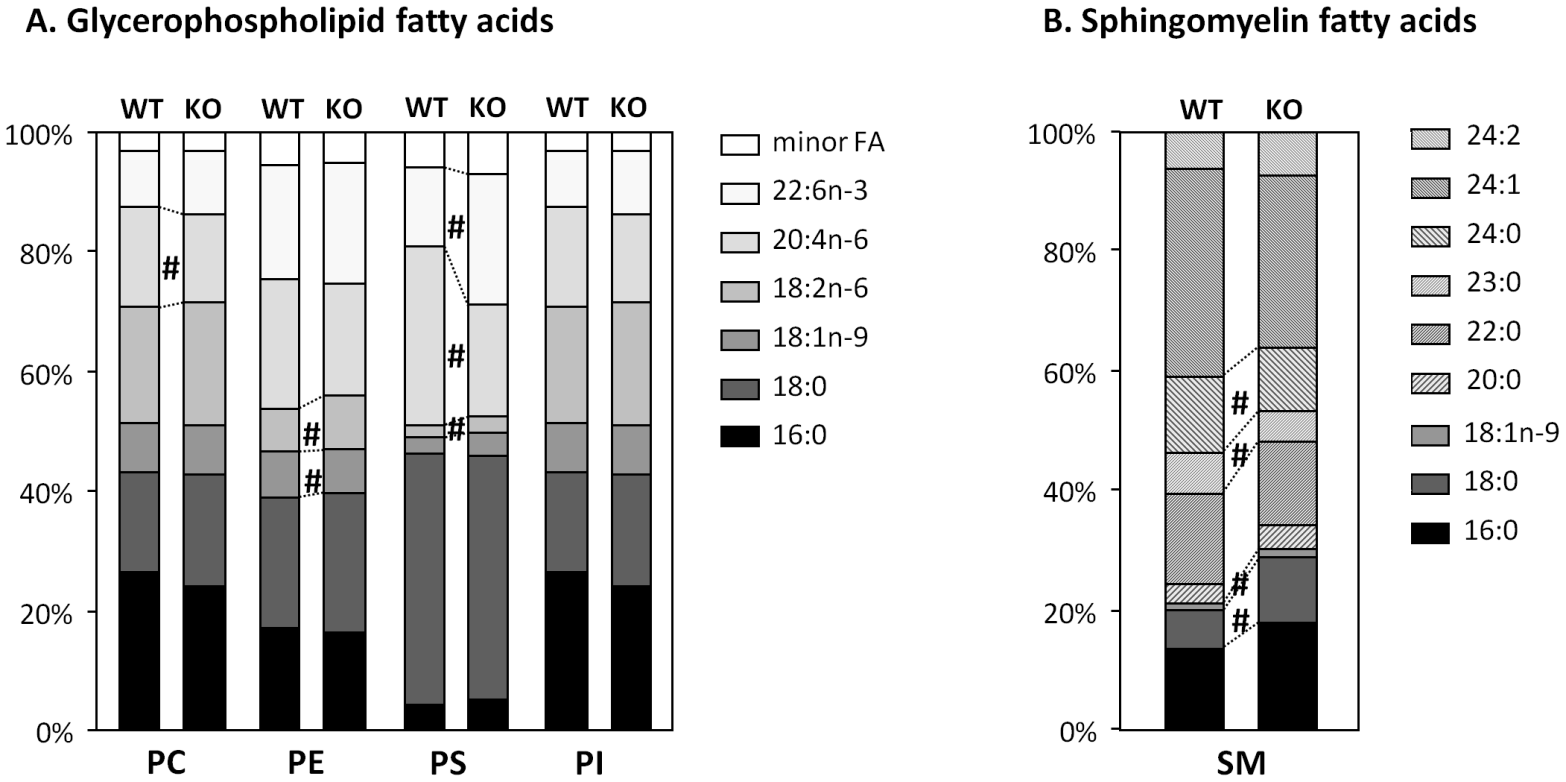
E2F2 gene deletion modifies the acyl diversity of major phospholipid classes. Partial hepatectomy was performed on E2F2^+/+^ (wild-type, WT) and E2F2^-/-^ (KO) mice, and were sacrificed 48 hours later. Quiescent (0-h) and regenerating (48-h) livers were harvested and homogenized and lipids extracted from homogenates. The fatty acid composition of phosphatidylcholine (PC), phosphatidylethanolamine (PE), phosphatidylserine (PS), phosphatidylinositol (PI) and sphingomyelin (SM) were determined by gas chromatography analysis after their separation by thin-layer-chromatography. Only the data values for quiescent livers are shown as profiles in regenerating livers are highly similar. Results are expressed as mol% of the total fatty acid in each phospholipid class and presented as means ± SD of two independent experiments using cohorts of 6 WT and 6 E2F2^-/-^ mice each. Statistical differences between the same tissue condition of different genotypes are denoted by # *P*≤0.05 (unpaired, 2-tailed Student's *t*-test).

**Figure 4:**
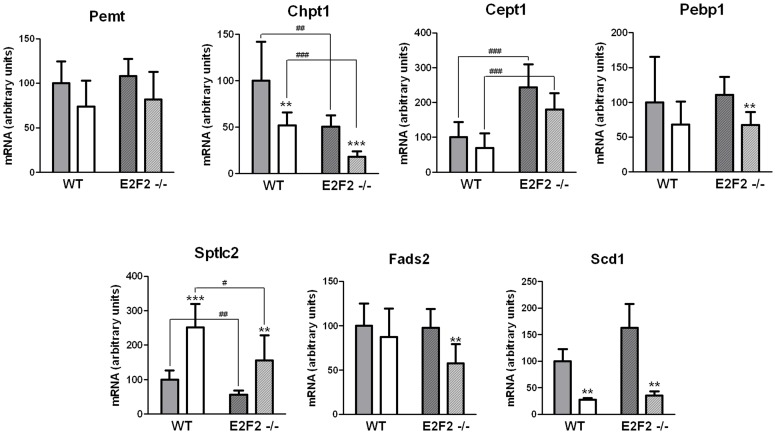
E2F2 gene deletion promotes changes in transcript expression of selected genes involved in phospholipid and fatty acid metabolism. Partial hepatectomy was performed on E2F2^+/+^ (wild-type, WT) and E2F2^-/-^ mice, and were sacrificed 48 hours later. Quiescent (0-h, solid bars) and regenerating (48-h, open bars) livers were harvested and total RNA from each mouse was examined in triplicate by quantitative real-time PCR for the following genes: Pemt, phosphatidylethanolamine N-methyltransferase; Chpt1, choline phosphotransferase 1; Cept1, choline/ethanolamine phosphotransferase 1; Pebp1, phosphatidylethanolamine binding protein 1; Sptlc2, serine palmitoyltransferase, long chain base subunit 2; Fads2, fatty acid desaturase 2; Scd1, stearoyl-CoA desaturase 1. Relative transcript abundance was calculated for each mouse using a normalization factor computed by Genorm software for pyruvate carboxylase, transferrin receptor and vascular endothelial zinc finger containing factor 1 mRNAs. Values are referred to 0-h WT mice samples (100%). Results are presented as means ± SD (n = 8 mice per group), except for Scd1 transcript levels, which were estimated in 3 pools of eight mice each from each genotype (n = 3). Statistical differences between regenerating and quiescent liver of a genotype are denoted by ** *P*≤0.01, *** *P*≤0.001, and between the same tissue condition of different genotypes are denoted by # *P*≤0.05, ## *P*≤0.01, ### *P*≤0.001 (unpaired, 2-tailed Student's *t*-test).

E2F2^+/+^ (wild-type, WT) and E2F2^-/-^ mice used in the described experiments are progeny of the first-generation backcross of the E2F2 null allele onto the C57Bl6 genetic background (mixed C57B16∶129Sv strain). All the mice were housed and bred in our mouse colony at the University of the Basque Country animal facility, and were genotyped as previously described [21], [22]. In total, 48 eight- to ten-week old female mice were used in these studies: 24 WT mice and 24 E2F2^-/-^ mice. We used three cohorts of 8 animals per genotype making a total of 24 E2F2^-/-^ mice and their respective littermate controls (n = 24) for the microarray analysis (Tables S1, S2 and S3). Six out of the eight mice were randomly selected for lipid composition analysis. Thus, values in Figures 1 and 2 are the result of three independent experiments using cohorts of 6 E2F2^-/-^ and 6 WT mice each, and values in Figure 3 and Tables 1 and 2 are the result of two independent experiments using cohorts of 6 E2F2^-/-^ and 6 WT mice each. Gene expression analysis by quantitative real-time PCR was done in 8 mice per genotype except for Scd1 transcript levels, which were estimated in the 3 pools of 8 mice each from each genotype (Figure 4).

**Table 1:** Fatty acid composition of total lipids in wild-type (WT) and E2F2^-/-^ mice liver before (0-h) and 48 hours (48-h) post-partial hepatectomy.

Fatty acid	WT mice liver								E2F2^-/-^ mice liver								
	0-h			48-h					0-h			48-h					
	%			%					%			%					
14:0	0.3	±	0.1	0.8	±	0.1			0.2	±	0.04	0.7	±	0.1			
14:1 n-9	0.04	±	0.02	0.1	±	0.04		*	0.04	±	0.01	0.1	±	0.03			
15:0	0.1	±	0.01	0.1	±	0.02			0.1	±	0.01	0.1	±	0.02			
15:1 n-9	0.01	±	0.011	0.1	±	0.01		*	0.03	±	0.005	0.1	±	0.01			
16:0	20.9	±	0.8	19.7	±	0.5		*	20.8	±	1.3	17.9	±	0.6			
16:1 n-9	2.1	±	0.4	5.2	±	1.5		*	2.2	±	0.3	4.5	±	0.6			
17:0	0.3	±	0.05	0.2	±	0.02		*	0.3	±	0.02	0.1	±	0.01			
17:1 n-9	0.2	±	0.01	0.3	±	0.03			0.2	±	0.01	0.2	±	0.1			
18:0	10.9	±	1.3	3.6	±	1.0		*	10.6	±	1.4	3.8	±	0.6			
18:1 n-9	16.7	±	1.2	27.3	±	0.8		*	19.5	±	0.5	26.5	±	0.7			
18:2 n-6	23.8	±	1.9	30.8	±	0.5		*	23.0	±	2.2	33.5	±	0.2	#		
18:3 n-6	0.4	±	0.1	0.7	±	0.2			0.4	±	0.1	0.6	±	0.2			
18:3 n-3	0.8	±	0.2	1.4	±	0.3			0.8	±	0.2	1.7	±	0.2			
20:2 n-6	0.3	±	0.03	0.6	±	0.1			0.4	±	0.1	0.5	±	0.1			
20:3 n-9	0.5	±	0.05	0.7	±	0.02			0.5	±	0.1	0.7	±	0.1			
20:3 n-6	1.0	±	0.1	0.9	±	0.2			1.0	±	0.2	0.8	±	0.1			
20:4 n-6	11.8	±	1.1	3.4	±	0.7		*	11.1	±	1.3	3.9	±	0.7			
20:5 n-3	0.6	±	0.2	0.3	±	0.02			0.8	±	0.1	0.4	±	0.1			
22:4 n-6	0.2	±	0.1	0.03	±	0.01			0.2	±	0.1	0.03	±	0.02			
22:5 n-6	0.9	±	0.1	0.5	±	0.1			0.8	±	0.1	0.5	±	0.1			
22:5 n-3	0.9	±	0.2	0.5	±	0.1			0.8	±	0.1	0.3	±	0.1			
22:6 n-3	7.2	±	0.8	3.0	±	0.8		*	6.3	±	0.4	3.0	±	0.6			
SFA	32.5	±	2.7	24.4	±	1.3		*	32.0	±	2.2	22.6	±	1.6			
MUFA	19.1	±	0.7	32.9	±	1.4		*	21.9	±	1.7	31.4	±	2.4			
PUFA n-6	38.1	±	4.0	36.4	±	1.3			36.5	±	3.4	39.3	±	1.8			
PUFA n-3	9.5	±	0.7	5.1	±	0.4		*	8.7	±	1.3	5.5	±	0.4			
n-6/n-3	4.0	±	0.2	7.1	±	0.9			4.2	±	0.4	7.1	±	0.7			
Unsat. Index	181.6			145.0					171.8			166.7					
C16	23.0			24.9					23.0			22.4					
C18	52.6			63.8					54.3			66.1					
C20	14.2			5.9					13.8			6.3					
C22	9.2			4.0					8.1			3.8					

The results are expressed as a percentage of the fatty acids measured in the corresponding total lipid sample and are the average ± SD of two independent experiments using cohorts of 6 E2F2^+/+^ (wild-type, WT) and 6 E2F2^-/-^ mice each. SFA, saturated fatty acids; MUFA, monounsaturated fatty acids; PUFA, polyunsaturated fatty acids. Student's *t*-test for paired data: * *P*≤0.05 for comparisons of 48-h *vs* 0-h within the same genotype; # *P*≤.05 for comparisons of the same condition between E2F2^-/-^ and WT groups.

**Table 2:** Fatty acid composition of triacylglycerols in wild-type (WT) and E2F2^-/-^ mice liver before (0-h) and 48 hours (48-h) post-partial hepatectomy.

Fatty acid	WT mice liver								E2F2^-/-^ mice liver					
	0-h			48-h					0-h		48-h			
	%			%					%		%			
14:0	0.65	±	0.18	0.62	±	0.10		0.50	±	0.04	0.82	±	0.17	
14:1 n-9	0.07	±	0.04	0.07	±	0.04		0.05	±	0.02	0.12	±	0.03	
15:0	0.13	±	0.01	0.1	±	0.02		0.12	±	0.03	0.13	±	0.02	
15:1 n-9	0.04	±	0.011	0.06	±	0.02		0.04	±	0.02	0.08	±	0.03	
16:0	21.5	±	0.85	16.2	±	0.59	*	21.1	±	0.95	17.9	±	0.45	
16:1 n-9	3.98	±	0.36	3.10	±	0.82		3.68	±	0.33	5.20	±	0.39	
17:0	0.17	±	0.07	0.15	±	0.06		0.19	±	0.02	0.12	±	0.04	
17:1 n-9	0.25	±	0.01	0.23	±	0.03		0.26	±	0.01	0.31	±	0.10	
18:0	2.14	±	0.43	2.92	±	0.75		2.06	±	0.35	1.87	±	0.60	
18:1 n-9	26.5	±	1.1	29.0	±	0.7		31.1	±	0.5 #	28.1	±	0.8	
18:2 n-6	33.5	±	1.3	34.5	±	0.6	*	31.3	±	1.4	35.1	±	0.6	
18:3 n-6	0.81	±	0.10	0.75	±	0.25		0.57	±	0.16	0.61	±	0.12	
18:3 n-3	1.87	±	0.21	1.20	±	0.29		1.66	±	0.24	2.03	±	0.17	
20:2 n-6	0.47	±	0.08	1.05	±	0.10		0.67	±	0.09	0.56	±	0.11	
20:3 n-9	0.60	±	0.09	1.20	±	0.13		0.74	±	0.10	0.73	±	0.15	
20:3 n-6	0.78	±	0.14	1.59	±	0.29		0.84	±	0.16	0.86	±	0.13	
20:4 n-6	2.67	±	0.72	2.42	±	0.55		2.12	±	0.58	2.20	±	0.47	
20:5 n-3	0.40	±	0.22	0.26	±	0.05		0.34	±	0.06	0.30	±	0.10	
22:4 n-6	0.64	±	0.09	0.87	±	0.04		0.50	±	0.07	0.43	±	0.02##	##
22:5 n-6	0.34	±	0.05	0.49	±	0.02		0.29	±	0.06	0.24	±	0.04	#
22:5 n-3	0.53	±	0.20	0.62	±	0.03		0.37	±	0.08	0.33	±	0.05	#
22:6 n-3	2.01	±	0.09	2.48	±	0.20		1.63	±	0.13	1.94	±	0.46	
SFA	24.6	±	1.7	20.0	±	1.3		23.9	±	1.2	20.9	±	1.4	
MUFA	30.7	±	0.5	32.4	±	1.2		35.0	±	1.3 #	33.7	±	2.0	
PUFA n-6	39.2	±	4.0	41.7	±	1.1		36.3	±	2.6	40.1	±	1.3	
PUFA n-3	4.8	±	0.6	4.6	±	0.5		4.0	±	1.0	4.6	±	0.6	
n-6/n-3	8.2	±	1.3	9.1	±	0.9		9.1	±	0.4	8.7	±	0.7	
Unsat. Index	141			150				134			142			
C16	25.4			19.3				24.7			23.1			
C18	64.8			68.4				66.6			67.7			
C20	4.9			6.5				4.7			4.6			
C22	3.5			4.5				2.8			2.9			

The results are expressed as a percentage of the fatty acids measured in the corresponding triacylglycerol sample and are the average ± SD of two independent experiments using cohorts of 6 E2F2^+/+^ (wild-type, WT) and 6 E2F2^-/-^ mice each. SFA, saturated fatty acids; MUFA, monounsaturated fatty acids; PUFA, polyunsaturated fatty acids. Student's t-test for paired data: *P≤0.05 for comparisons of 48-h *vs* 0-h within the same genotype; # *P*≤0.05 and ## *P*≤0.01 for comparisons of the same condition between E2F2^-/-^ and WT groups.

Partial (70%) hepatectomy was performed under isofluorane anesthesia according to the Higgins and Anderson method, as recently described [11]. To minimize inter-variability of regeneration outcomes, the surgeries were done by the same investigator. The animals studied in this work had no apparent suprahepatic vena cava stenosis. Standard chow diet and water were provided ad libitum and animals left to recover for 48 hours in the original thermostatized cage without the use of postoperative analgesia.

Confirming previous observations [5], at 48-h following PH steatosis reached grade 2 (affecting 33–66% hepatocytes) and liver mass restoration was similar in the sex- and age-matched WT and E2F2^-/-^ mice cohorts used here. Liver mass on day 2 doubled in the two groups, rising from ∼30% to ca. 68% of the initial pre-PH hepatic index, which was also similar in the two experimental groups (initial liver/body weight ratio was 0.0435±0.0008 for WT mice and 0.0445±0.0008 for E2F2^-/-^ mice).

Animal experiments were approved by the University of the Basque Country ethical committee CEEA in accordance with the guidelines of European Research Council for animal care and use. Liver aliquots were immediately homogenized for lipid analysis or frozen in liquid N_2_ and stored at −80°C for mRNA analysis.

### Hepatic lipid analysis

Livers (200 mg) were homogenized in ice-cold phosphate-buffered saline (10 mM sodium phosphate, pH 7.2, 150 mM sodium chloride) immediately after surgery (0-h) or euthanasia (48-h) using a Polytron homogenizer (Brinkmann Instruments, Westbury, NY). Protein content in homogenates was analyzed using a bicinconinic acid-based commercial reagent (Bio-Rad). Lipids were exhaustively extracted from homogenates as described before [28], and different aliquots were taken for either the determination of TAG, or total lipid phosphorus and FA composition or for the separation of PL by thin-layer-chromatography. Aliquots were stored at −80°C under N_2_ until analysis.

TAG was quantified by commercial kits used in clinical settings (Boehringer Mannheim Gmbh, Mannheim, Germany), according to the manufacturer's instructions. Phosphorus content of PL classes was determined after isolation by thin-layer-chromatography using chloroform∶methanol∶acetic acid∶water (50∶37∶3.5∶2, v/v) as the solvent system in saturated chambers [29]. Phospholipid spots were visualized by iodine vapor staining and scraped for phosphorus analysis [30]. Phospholipid classes for FA analysis were isolated using a chloroform∶methanol∶amonium (65∶25∶5, v/v) and chloroform∶acetone∶methanol∶acetic acid∶water (30∶40∶10∶10∶3, v/v) two dimensional solvent system. Phospholipid spots were located after spraying the plates with dichlorofluorescein in methanol, scraped and eluted with chloroform/methanol/acetic acid/water (50∶39∶1∶10, v/v) [31]. Eluted sphingomyelin (SM) was subjected to three successive steps for purification: mild alkaline treatment (0.5 N NaOH in anhydrous methanol at 50°C for 10 min), re-extraction by addition of chloroform and 0.5 N HCl to the methanolic phase and thin-layer-chromatography separation using chloroform∶methanol∶acetic acid∶0.15 M NaCl (50∶25∶8∶2.5, v/v) [32].

FA composition of lipids was determined by gas-chromatography of their FA methyl ester derivatives prepared by keeping the lipid samples overnight at 45°C under N_2_ in the presence of anhydrous methanol containing 0.5 N H_2_SO_4_ in Teflon lined, screw capped tubes [33]. Before gas-chromatography FA methyl esters were purified using pre-washed silica gel G thin-layer-chromatography plates with methanol∶ether (75∶25, v/v) and hexane∶ether (95∶5, v/v) as the solvent system. FA methyl esters were visualized on the plates using dichlorofluorescein and recovered from the silica after thorough mixing and partition between water∶methanol∶hexane (1∶1∶1, v/v/v) in three successive hexane extractions. A Varian 3700 gas chromatograph equipped with two (2 m×2 m) glass columns packed with 10% SP 2330 on Chromosorb WAW 100/120 (Supelco, Inc.) was used. The column oven temperature was programmed from 155°C to 230°C at a rate of 5°C/min, and then kept at the upper temperature for about 10 min. Injector and detector temperatures were set at 220 and 230°C, respectively, and N_2_ (30 ml/min) was the carrier gas. The FA peaks were detected with flame ionization detectors, operated in the dual-differential mode, and quantified by electronic integration (Varian Workstation).

### RNA preparation and gene expression analysis by quantitative real-time PCR

Total RNA was extracted from liver tissue (100 mg) using TRIzol reagent (Invitrogen), purified (RNeasy Mini kit, Qiagen), DNase I-treated (Invitrogen) and re-purified according to the manufacturers' instructions. The purity and concentration of RNA was determined with a NanoDrop ND-1000 UV-Vis spectrophotometer (NanoDrop Technologies). The 260/280 nm absorbance ratio of samples was in the 1.8–2.1 range. RNA integrity was further assessed by running samples in 1% agarose gel electrophoresis. cDNA was synthesized from 3.6 µg total RNA using the SuperScript III First-Strand Synthesis System for reverse transcription PCR kit (Invitrogen) according to the manufacturer's recommendations. For quantitative real-time PCR analyses of target mRNA levels TaqMan Gene Expression Assays (Applied Biosystems) were used. References of TaqMan assays were the following: Mm00839436_m1 for phosphatidylethanolamine N-methyltransferase (Pemt, GI:33667035); Mm00772290_m1 for stearoyl-CoA desaturase 1 (Scd1, GI:118130513); Mm01208299_g1 for fatty acid desaturase 2 (Fads2, GI:9790070); Mm00522694_m1 for choline phosphotransferase 1 (Chpt1, GI:38604066); Mm01200029_g1 for choline/ethanolamine phosphotransferase 1 (Cept1, GI:142365741); Mm02601848_g1 for phosphatidylethanolamine binding protein 1 (Pebp1, GI:84794551); and Mm00448878_m1 for serine palmitoyltransferase, long chain base subunit 2 (Sptlc2, GI:142366849). The relative quantities of each gene were determined by the ΔΔCt method. Normalization was performed using normalization factors computed by GeNorm for pyruvate carboxylase (Pcx, Mm00500992_m1), transferrin receptor (Tfrc, Mm00441941_m1) and vascular endotelial zinc finger containing factor 1 (Vezf1, Mm00497288_m1) mRNAs, as detailed previously [5].

### Statistical analysis

Lipid composition results are presented as means ± SD. Statistical analyses were performed using GraphPad Prism v5.02 for Windows (GraphPad Software, San Diego, CA). Significance was determined by the Student's *t*-test for unpaired data for comparisons between two individual data groups or for paired data for comparisons of FA composition values. A *P*≤0.05 value was considered statistically significant unless otherwise stated.

## Results

### Deregulated expression of lipid metabolism genes in E2F2-deficient liver

To better understand the connection between E2F2 activity and lipid metabolism, we re-evaluated microarray raw data of quiescent E2F2^+/+^ and E2F2^-/-^ mice liver transcriptomes that we had gathered in a previous study [5]. These data have been deposited in the ArrayExpress repository (www.ebi.ac.uk/arrayexpress) under accession number E-MEXP-1413. The microarray analysis produced an unexpectedly large number of deregulated codes (2915), 59.5% of which were upregulated and 40.5% of which were downregulated in the liver of E2F2^-/-^ compared with WT mice (*P*≤0.01) (Table S1). Gene Ontology (GO) analysis of the deregulated genes identified eleven GO terms with a significant enrichment according to Bonferroni (*P*≤0.05) and Benjamini-Hochberg (*P*≤0.01) multiple testing corrections (Table S2). Remarkably, nine GO Biological Processes were linked to core metabolic pathways in which “lipid metabolic process” was the second most enriched GO term after “transport”, showing a false discovery rate close to zero. A set of sequences encoding 208 lipid metabolism-associated proteins (73.1% upregulated and 26.9% downregulated) was identified as E2F2-dependent (Table S3). We found alterations in mutant mice compared to control in the expression of essential genes involved in the following processes: mitochondrial (i.e. *Acad10, Acsl1, Atp5g3, Abcd2, Cpt2, Slc25a24, Ucp2*) and peroxisomal (i.e. *Pecr, Slc27a5*) FA oxidation; FA synthesis (i.e. *Acot11, Faah, Fads1, Fads2, Ffar2, Scd1*) and precursor use of FA (i.e. *Cyp2c37, Ptgs2*); glycerolipid metabolism (i.e. *Abhd4, Acaa1b, Atp8a1, Cav1, Cds2, Chka, Chpt1*, *Etnk2, Far1, GK2, Lpcat3, Lpin3, Ocrl, Pcyt2, Ptdss1, Ptdss2*, phospholipases as *Ddhd2, Enpp2*, *Ppap2a, Ppap2b* and *Pnpla6*, and phosphoinositides signaling related genes); sphingolipid metabolism (i.e. *Naaa, Sptlc3, Smpd3, Sgms1, St3gal6, St6galnac6, Sult1e1, B3galt5, Ugcg*), as well as genes involved in steroid metabolism and bile secretion (i.e. *Abcb4, Slc10a7, Soat1, Srebf2*) and plasma lipid transport and metabolism (i.e. *Afp, Apob, Apobec2, Abca2, Lrp1, Lrp3, Sulf2*). These genetic alterations argue that mice lacking E2F2 could display abnormalities in hepatic lipid balance. These findings prompted us to further investigate the role of E2F2 in liver lipid homeostasis *in vivo*, and to examine if the PL and FA composition of quiescent and regenerating (48-h) liver tissue was dependent on E2F2 gene activity.

### Hepatic phospholipid composition is altered in E2F2-deficient mice

Mouse liver total TAG, PL and protein content (mg per g of liver) were assessed in quiescent state and after PH (Figure 1). Regenerating WT liver tissue was richer in TAG (6-fold increase) and protein (0.2-fold increase) than quiescent WT liver, whereas the concentration of PL per gram of liver remained constant. Similar results were obtained in E2F2^-/-^ mice, suggesting that the shift from PL to TAG as the dominant lipid component in the tissue ongoing regeneration is E2F2-independent.

To evaluate whether E2F2 activity has an effect on PL distribution we determined the relative proportion of each PL class by measuring the phosphorous content of the major PL classes after thin-layer-chromatography separation, both in quiescent and regenerating livers (Figure 2). Liver PL distribution in quiescent liver was markedly different between WT and E2F2^-/-^ mice. PC and PE were the major PL classes in quiescent WT liver, and accounted for ∼52% and ∼22% of PL content, respectively. Remarkably, we found that PC levels were lower (30%) and that PE levels were significantly higher (69%) in quiescent E2F2^-/-^ liver tissue relative to WT counterparts. Consequently, the hepatic PC/PE ratio in quiescent tissue was much lower in E2F2^-/-^ mice (0.987) compared to E2F2^+/+^ mice (2.375), indicating that E2F2 gene activity contributes to sustain the homeostasis of the two major membrane phospholipid classes when cells do not proliferate. Likewise, the ratio of choline-containing PL (PC+LPC+SM) versus amino-containing PL (PE+PS) was lower in E2F2^-/-^ mice liver (2.138) compared to WT (1.002). 48 hours after partial hepatectomy, PC and PE percentages (Figure 2) as well as the PC∶PE ratio in WT livers changed significantly relative to quiescence resembling percentages found in quiescent E2F2^-/-^ liver samples. These parameters remained unchanged in regenerating E2F2^-/-^ tissue, indicating that no further proliferation-driven quantitative adjustments were required in the absence of E2F2. Altogether our results indicate that *E2F2* gene deletion not only alters the PL homeostasis in Q_0_/Q_1_ (resembling the PC∶PE ratio of proliferating WT tissue) but also constrains the normal response of the liver to regeneration-associated signals that control PC and PE homeostasis. By contrast to major PL classes, neither E2F2 deficiency nor regeneration signals affected significantly the relative abundance of the minor amino-phospholipid phosphatidylserine (PS), the choline-phospholipids lysophosphatidylcholine (LPC) and sphingomyelin (SM), phosphatidylinositol (PI), cardiolipin (diphosphatidylglycerol, DPG) or the intermediate phosphatidic acid (PA).

### Fatty acid profiling of WT and E2F2-deficient mouse livers

To test whether E2F2 gene is required for normal esterification of FA into lipids, and to maintain homeostasis of liver lipids, we determined the FA composition of total lipids 48 hours after PH and compared to the pre-PH composition in WT and E2F2^-/-^ mouse liver samples (Table 1). The global hepatic FA composition was consistently similar in quiescent WT and E2F2^-/-^ mice and the regeneration induced changes were E2F2-independent. The most abundant FA in the liver of the mouse strains used here were 18∶1 (oleic acid) of the n-9 family, 18∶2 (linoleic acid) and 20∶4 (arachidonic acid, AA) of the n-6 family, 22∶6 (docosahexaenoic acid, DHA) of the n-3 family, and the saturated fatty acids (SFA) 16∶0 (palmitic acid) and 18∶0 (stearic acid). At 48-h post-PH, SFA, mostly 18∶0, decreased (WT, 25%; E2F2^-/-^, 29%) and the monounsaturated fatty acids (MUFA, 18∶1n-9 and 16∶1n-9) increased (WT, 72%; E2F2^-/-^, 43%), whereas total n-6 polyunsaturated fatty acids (PUFA) did not change. Interestingly, the marked decrease in the pro-inflammatory AA (WT, 72%; E2F2^-/-^, 65%) was compensated by the increase in its precursor linoleic acid (WT, 30%; E2F2^-/-^, 45%). Anti-inflammatory n-3 PUFA decreased (WT, 47%; E2F2^-/-^, 37%) due to DHA reduction. Remarkably, in regenerating liver, C18 fatty acids represented nearly 65% of the fatty acids, and the proportion of products to precursor 20∶5+22∶6/18∶3n-3 and 20∶4/18∶2n-6 dropped to one fourth. Since the overall changes were similar in the two genotypes, it is possible that repression of elongase activity and activation of Δ9 desaturase activity during regeneration be independent of E2F2 activity. Only two of the parameters measured at 48-h post-PH were indeed sensitive to E2F2 loss: the rise in 18∶2n-6, which was 50% higher in the absence of E2F2, and the 20% decrease in unsaturation index, which was not apparent in E2F2^-/-^ mice.

The differences in the FA composition of total lipid in liver regeneration described above were consistent with previous reports on proliferating cells [34]. To determine if the changes observed in the total lipid FA composition reflected specific modifications in the FA species of one or more lipids, or if they were the consequence of the relative contribution of different amount of lipids to the total pool, we performed a comprehensive FA composition analysis of major liver lipids, namely TAG (Table 2) and the phospholipids PC, PE, PS, PI, and SM (Figure 3). Major fatty acids in TAG were 16∶0, 18∶1n-9 and 18∶2n-6. Interestingly, the only changes induced by PH were modest decreases in the percentage of 16∶0 and increases in 18∶2n-6, indicating that the FA composition of newly synthesized TAG that accumulate massively in lipid droplets was almost identical to that of pre-existing TAG. Since 18∶1n-9 and 18∶2n-6 are major TAG-FA, the six-fold increase in the content of TAG at 48-h following PH was likely accounting for the increase observed in these two FA in total lipids. Data indicate that E2F2 has a minor influence on the hepatic TAG-FA composition, either in quiescence, where it affects the 18∶1 contribution (∼25% higher in mice that lack E2F2) and the MUFA proportion, or in regeneration, where it affects the low abundant 22C fatty acids.

The FA composition of PL classes was remarkably similar before and 48 hours after PH, and E2F2 deletion caused similar changes in quiescent and regenerating tissue (Figure 3 and data not shown). Six fatty acids (16∶0, 18∶0, 18∶1n-9, 18∶2n-6, 20∶4n-6 and 22∶6n-3) accounted for ∼95% of total FA chains in liver glycerophospholipids. Analysis of the phospholipid classes revealed a significant reduction in PC and PS 20∶4n-6 and in PE 18∶1n-9 and a significant increase in PE and PS 18∶2n-6, and PS 22∶6n-3 in E2F2^-/-^ compared to WT. The singular FA composition of hepatic SM was slightly different in mutant mice, with exchange of very-long-chain saturated 24∶0 and 23∶0 for 18∶0 and 18∶1n-9. Our results indicate that, besides the abnormal low proportion of PC to PE in quiescence, constitutive E2F2 deletion in mice leads to reorganization of the molecular species of hepatic PC, PE, PS and SM. Since these changes are sustained during normal proliferation of liver cells, loss of E2F2 could have a potential impact on multiple membrane-dependent cell processes.

### E2F2 gene deletion deregulates the expression of genes involved in phospholipid synthesis

**Figure 5:**
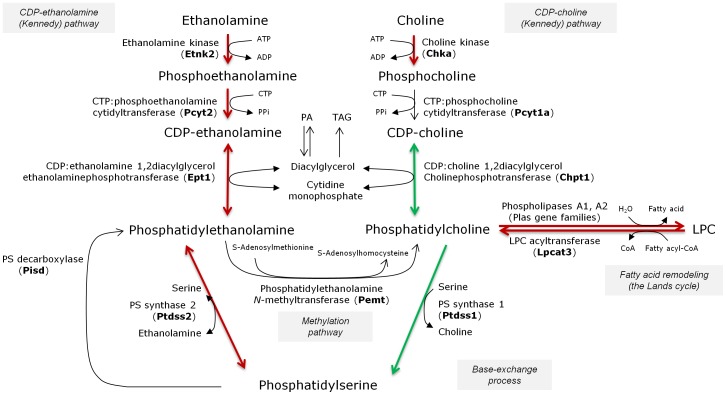
Scheme representing E2F2 as a regulator of the major metabolic pathways that generate phosphatidylcholine and phosphatidylethanolamine in the mammalian liver. The name in bold between parentheses corresponds to the gene coding for the dominant isoform in adult liver. Constitutive deletion of E2F2 gene promotes increased phosphatidylethanolamine (PE) relative content, a low phosphatidylcholine (PC):PE ratio, increased expression of genes required for PE formation and deregulated expression of some genes involved both in the Kennedy pathway for PC synthesis and the deacylation:reacylation arm of PC turnover. Such permanent decrease in the PC:PE ratio may impact phospholipid-related biological responses. CTP, cytidinetriphosphate; CDP, cytidinediphosphate; LPC, lysophosphatidylcholine, PA, phosphatidic acid; TAG, triacylglycerol. The Lands (deacylation-reacylation) cycle consists of phospholipases A_2_ (or A_1_) and acyl:CoA lysophospholipid acyltransferases. We only show here the deacylation/reacylation of phosphatidylcholine. Transcript upregulations are shown in red and downregulations in green.

To further investigate the role of E2F2 on the relative proportion of PC and PE levels and on elongase/desaturase activity, we determined by quantitative real-time PCR the transcript expression levels of several genes involved in lipid metabolism that appeared deregulated in our initial microarray analysis [5]. The selected genes were *Pemt*, *Chpt1*, *Cept1*, *Pebp1*, *Sptlc1*, *Fads2* and *Scd1* (Figure 4). Our findings indicate that *Chpt1*, the gene encoding the enzyme that synthesizes PC through the CDP-choline pathway, is markedly down-regulated in quiescent E2F2^-/-^ mice liver compared with WT mice, and additionally repressed in regenerating liver (see Figure 5 for a schematic representation of the major metabolic pathways that generate PC and PE in the liver). No differences were observed for any experimental condition and genotype in *Pemt* expression, responsible for the liver-exclusive methylation of PE to form PC. However, the *Cept1* gene, which is involved in the biosynthetic pathway leading to PE was upregulated in mutant mice and unaffected by regeneration. Notably, *Sptlc2*, responsible for sphingosine synthesis was downregulated by E2F2 loss and upregulated in regenerating tissue in the two strains. Conversely, *Scd1* was dramatically downregulated in regenerating tissue in the two strains. Downregulation of *Pebp1* and *Fads2* is associated with proliferation only in the absence of E2F2, and the hepatectomy-promoted decrease in *Chpt1* and *Scd1* expression is more marked in E2F2 deficient as compared with WT liver, suggesting that E2F2 may modulate the transcriptional repression induced by regenerative signals on certain genes.

## Discussion

The studies reported here were designed to characterize the *in vivo* role of E2F2 transcription factor in liver lipid homeostasis. This question was raised by our own previous data that implicated E2F2 in the timely mobilization of the LDs accumulated during the post-PH regenerative process [5]. A subsequent finding from our group also showed that the expression of numerous genes involved in the hepatic “lipid metabolism process” was deregulated in the liver of E2F2 nullizygous mice in quiescence (Tables S2 and S3). Here we have shown that E2F2 sustains normal hepatic PC to PE ratios and appears to function as a transcriptional regulator of genes required for the synthesis of these two major phospholipids, contributing also to the acylic diversity of membrane glycerolipids and sphingomyelins in proliferating and non-proliferating liver tissue.

A PC to PE ratio close to 2.5 is crucial for normal membrane function, and variations of this proportion are commonly associated with cell proliferation [35], but also with disease [36]. Lipids in biological membranes are asymmetrically distributed across the bilayer with most of the amine-containing PE (and PS) localized in the inner leaflet and the choline-containing PC (and LPC and SM) in the outer leaflet [37]. But lipid asymmetry cannot be maintained when PC is insufficient, which may trigger apoptosis in cells [38], [39], or if the PC/PE ratio decreases, as is commonly found in patients clinically diagnosed with non-alcoholic steatohepatitis (1.2 versus 2.5 in normal controls) or in animal models of choline deficient diet-induced steatohepatitis [36]. Contrary to the changes observed in livers from these animals (decreased content of PC levels and no change or a minor increase of PE) [36], [38], quiescent E2F2^-/-^ livers contained increased abundance of PE and no change or a tendency to decrease of PC (Figure 2). Accordingly, the hepatic PC to PE ratio (PC+LPC+SM to PE+PS ratio) drops markedly (0.987 (1.002) versus 2.375 (2.138) in WT controls) in response to the loss of E2F2. Following a canonical way of thinking, this suggests that some surface PC (a cylindrical, bilayer molecule) might be replaced by the inverted cone-shaped PE in the liver membranes of E2F2^-/-^ mice, potentially leading to defective curvature and packing of cell membranes as well as defective activity of integral proteins [37]. As part of the physiological events culminating in cell division [40], cell membranes of proliferating cells have a PC∶PE ratio around 1 [35], which returns to quiescent levels after cytokinesis [40]. However, this phenotype is chronically displayed by liver membranes in cells that are in G_0_ in E2F2^-/-^ mice, suggesting that the loss of functional E2F2 could potentially affect multiple yet undetermined membrane-dependent cell processes. Notably, GO analysis of deregulated hepatic genes in E2F2^-/-^ mice revealed that the biological process most robustly affected by the loss of E2F2 is “transport”, having a false discovery rate and Benjamini and Bonferroni *P* values equal to 0 (Table S2).

Phospholipid metabolism and its regulation are remarkably complex. In mammalian liver, PC is made from choline via the CDP-choline (Kennedy) pathway for *de novo* synthesis, the Lands cycle for remodeling of the FA composition of PC species [41], [42] and the liver-exclusive PE *N*-methyltransferase (PEMT)-driven methylation of PE [43]–[45]. In the Kennedy pathway, choline is activated sequentially with ATP and CTP and transferred to diacylglycerol to form PC mainly by the enzymes encoded by *Chka*, *Pcyt1a* and *Chpt1*. The Kennedy pathway and the Lands cycle operate in PE synthesis using analogous reactions to PC synthesis. A third PE source is mitochondrial PS decarboxylation, while subsequent base-exchange reactions catalyzed by PS synthase between PC (*Ptdss1*) or PE (*Ptdss2*) and serine generate PS. PS synthase 2 can also work in reverse producing PE from PS [45] (Figure 5). In line with the increased levels of PE in E2F2^-/-^ (Figure 2), we found upregulated expression of metabolic transcripts governing the *de novo* synthesis of PE (*Cept1* in Figure 4 and *Etnk2* in Table S3) in mice lacking E2F2, suggesting that their transcription is repressed by E2F2 in normal liver. Also loss of E2F2 alters choline metabolism genes and E2F2 seems to regulate the expression of enzymes involved in the initial and final steps of the CDP-choline pathway in opposing ways. PC synthesis is primarily controlled by CTP: phosphocholine cytidyltransferase α (CTα) translocation to membranes [43]–[45] and secondarily by transcriptional regulation of involved genes [41]. Work on transcriptional regulation of the CTα-encoding gene *Pcyt1a* revealed that it is repressed by the complex Sp1/E2F-retinoblastoma-histone deacetylase in quiescent mouse embryonic fibroblasts [46]. We found similar levels of *Pcyt1* expression in quiescent WT and mutant mice liver (Table S3), suggesting that E2F2 is unlikely to be the E2F family member contributing to transcriptional repression of the rate limiting enzyme of PC synthesis. Comparing with wild type mice, PC levels were unaffected or tended to decrease (Figure 2) whereas the transcript levels of the gene controlling choline kinase, *Chka*, increased four-fold in quiescent E2F2^-/-^ liver (Table S3). It is possible that phosphocholine is formed in excess but cannot be effectively transformed to PC when *E2F2* is disrupted. The opposite trends of changes in PC and PE levels during quiescence and the concerted downregulation of choline phosphotransferase and upregulation of ethanolamine phosphotransferase found in mice lacking E2F2 in quiescent and proliferating liver (Figure 4) support the concept that E2F2 transcription factor may affect the partitioning of common intermediates ATP, CTP and DAG towards *de novo* PE and PC synthesis, controlling cellular PE and PC provision.

A characteristic feature of liver regeneration is the accumulation of LDs in the tissue [1], [4]. We have shown that hepatic TAG levels are similar in WT and mutant mice either in quiescence or at 48h-post PH (Figure 1), suggesting that TAG synthesis is not under E2F2 control. Previous work has shown that the massive hepatic steatosis occurring during the first 3–4 days of regeneration in WT mice persisted over 7 days in E2F2 knockout mice [5], suggesting a defective mobilization of stored TAG in this condition. Hepatic LDs are metabolically active organelles [47], [48] composed of a core of neutral lipids, mainly TAG and cholesteryl esters, surrounded by a monolayer of PL in which LD-associated proteins are embedded. The biological properties and functions of these proteins is matter of intense study. PC has been identified as an essential molecule for LDs turnover, acting as a surfactant to prevent LDs coalescence and formation of large, lipase-resistant droplets [49]. While LDs are able to synthesize PC locally by the Lands cycle [50], during LDs expansion, the additional PC required to coat the enlarging surface is mostly provided by the Kennedy pathway [49]. Hence, the activity of the CDP-choline pathway dictates the rate at which the stored TAG may be accessible to intracellular lipases. Further studies will be required to quantify the LD-associated metabolites and enzymatic activities involved in TAG turnover during the regeneration period. We propose that the chronically limited PC supply in E2F2-deficent liver cells might contribute to the late mobilization of TAG during liver regeneration in E2F2^-/-^ mice.

Diversity of acyl moieties in cellular glycerophospholipids depends on the specificity of acyltransferases involved in *de novo* pathways [51] but mostly on the remodeling catalyzed by phospholipases A2, transacylases and lysophospholipid acyltransferases (for recent reviews see [42], [52]). E2F2 activity affects the acyl diversity of hepatic PC, PE, PS and SM (Figure 3) but not of PI, DPG (Figure 3), total lipids (Table 1) and TAG (Table 2). The major desaturases (*Scd1, Fads2*) and elongases (*Elovl6/Fasn*) are essential in supporting life-sustaining cellular processes. The global changes during regeneration are similar in the two strains (Figure 4 and [5]). It is intriguing why the proliferative liver maintains basically the same TAG fatty acid composition (Table 2) after a drastic increase in the amount of TAG being synthesized (Figure 1). A possibility is that TAG may serve as donors of DAG and/or fatty acyl chains for further incorporation into other lipids. Thus, the increased synthesis of different molecular species of TAG may be part of a metabolic adjustment to provide different FA for esterification into complex lipids. TAG might be then a metabolic transient store of glycerol and FA for *de novo* synthesis of PL. However, this may not be the case for FA with regulatory or precursor functions, whose changes in regeneration are associated with PL, as they are mostly incorporated in the remodeling cycle [42], [52]. Desaturases and elongases in mammalian cells transform the essential fatty acid 18∶3n-3 (linolenic acid) into the long-chain n-3 anti-inflammatory EPA and DHA and 18∶2n-6 (linoleic acid) into the pro-inflammatory AA, which are incorporated into lysophospholipids [42] prior to act as substrates for several oxygenase enzymes. PUFAs are thus major sources of fatty acid-derived lipid mediators [53], [54]. The balance between n-6 and n-3 PUFA families is particularly important during recovery from PH, as this is a pro-inflammatory situation. Indeed, the regenerative pressure led to similar global increases in the n-6/n-3 ratio and to the maintenance of constant proportions of essential fatty acids (Table 1). However, there were specific increases in PE and PS 18∶2n-6, decreases in PC and PS 20∶4n-6 and increases in PS 22∶6n-3 (Figure 4) in response to E2F2 loss in quiescence that were sustained during normal proliferation of liver cells, indicating that E2F2 is necessary for regulation of these fatty acids that play a significant role in physiopathological responses. Notably, the changes in FA composition of PS matched the acyl diversity of the PC and PE pools illustrating the complete reliance on their PC and PE substrates (Figure 5). The expression of numerous genes encoding proteins with phospholipase A_2_ activity sensitive to E2F2, such as *Lpcat3*, the main acyltransferase acting on lyso-PC, -PS and -PE acceptors in the liver [42], and *Plaa* (Table S3), also supports the role of E2F2 as modulator of PC, PE and PS reacylation.

Considering that steady-state levels of E2F2 increased after PH [27], that E2F2 activates the expression of the target genes required for S-phase entry [55]–[57] and that PC synthesis is active in the G_1_/S transition [58] it might be expected that E2F2 targets the genes involved in PC and PE metabolism in proliferating cells. However, findings in this and previous work [5], where a mouse strain carrying a loss-of-function mutation in E2F2 has been studied in quiescent and PH-associated liver regeneration, demonstrate that the *in vivo* effects of E2F2 deficiency, either in the PC∶PE ratio or in the expression levels of lipid genes, are more relevant in quiescent tissue. Paradoxically, the liver of E2F2^-/-^ mice harbors cells reluctant to enter S-phase after hepatectomy [5] but displays a membrane phenotype resembling an exit from G_0_/G_1_, regarding the low PC∶PE ratio and *Chka* upregulation, both markers for membrane proliferation [35], [59]. Overexpression of choline kinase and high levels of phosphocholine have been detected, in fact, in several tumors compared to surrounding non-cancerous tissue [60], [61]. It is worthwhile to note that examination of non-proliferating livers by microarray analysis has led to the identification of an unexpectedly elevated number of genes whose expression is deregulated (59.5% upregulated and 40.5% downregulated) in response to E2F2 deletion (Table S1). This supports dual functions for E2F2 in the liver, acting both as transcriptional repressor and activator, as was also found in other cell types [21], [23], [62]. E2F2 deletion resulted in a broad impact on core metabolic processes in normal liver (Table S2). Although, obviously, many of these effects may be indirect or due to compensation by members of the E2F family [63], our findings support the concept that E2F2 may have a role in metabolic homeostasis.

In conclusion, our data clearly demonstrate that the phenotype of adult liver carrying a constitutive E2F2 deletion includes deregulated expression of a set of 208 genes linked to lipid metabolism, leading to an aberrantly low proportion of PC to PE and abnormal acyl diversity in defined phospholipids that is maintained during regeneration. Further functional research is needed to determine the direct role of E2F2 on phospholipid metabolism in hepatocytes, the main functional cells of the organ, which will also provide additional insight into the complex relationship between membrane lipids and transcription factors mastering cell-cycle control.

## Supporting Information

Table S1 **E2F2 deletion deregulates the expression of a large number of sequences in quiescent liver.** Original data were extracted from the Affymetrix microarray data deposited in MIAME ArrayExpress database (www.ebi.ac.uk/arrayexpress) under accession code E-MEXP-1413. We used six RNA pools, three pools of E2F2^+/+^ (wild-type, WT) (n = 24) and three pools of E2F2^-/-^ (n = 24) mice genotypes. Each pool contained quiescent liver samples from 8 animals. Affymetrix chips and microarray data analysis were performed exactly as described in Ref. 5. TIGR MultiExperiment Viewer Mev version 4.1 (Institute of Genomic Research, Rockville, MD) was used for the statistical analysis of the microarray data. To identify the genes whose expression levels changed significantly in E2F2^-/-^ compared with WT mice we used Welch's t-test with a *P*≤0.01 significance level cut-off. After curation, 2915 sequences were found to elicit significant expression change in quiescent liver upon E2F2 loss; of them 1735 (59.5%) were upregulated and 1180 (40.5%) were downregulated.
(XLSX)

Table S2 **Overrepresented Gene Ontology functional categories in quiescent E2F2^-/-^ liver relative to wild-type controls.** ^a^Refers to the number of genes included in each overrepresented functional category.^ b^Refers to the percentage of genes detected in each functional category relative to the total number of genes included in that particular category. Functional classification of deregulated genes was made using FatiGO+, a public domain web tool for finding significant associations of Gene Ontology (GO) terms within groups of genes. Statistical significance was determined by Fischer's exact test and *P*-values were adjusted applying Benjamini-Hochberg (*P*≤0.01) and Bonferroni (*P*≤0.05) multiple testing correction.
(DOCX)

Table S3 **E2F2 deletion deregulates the expression of a set of genes involved in lipid metabolism in quiescent liver.** Genes (208) were extracted from the identified sequences listed in Table S1, categorized by our own criterion and shown in alphabetic order. Of them, 152 (73.1%) were up-regulated and 56 (26.9%) were down-regulated in the liver of E2F2 nullizygous mice in quiescence.
(DOCX)
