# Supplementary material for: The E2F2 Transcription Factor Sustains Hepatic Glycerophospholipid Homeostasis in Mice
Source: PLoS One. 2014 Nov 14;9(11):e112620. doi: 10.1371/journal.pone.0112620 (PMC4232400; doi:10.1371/journal.pone.0112620)
Supplement: Table S2 — Overrepresented Gene Ontology functional categories in quiescent E2F2-/- liver relative to wild-type controls. aRefers to the number of genes included in each overrepresented functional category. bRefers to the percentage of genes detected in each functional category relative to the total number of genes included in that particular category. Functional classification of deregulated genes was made using FatiGO+, a public domain web tool for finding significant associations of Gene Ontology (GO) terms within groups of genes. Statistical significance was determined by Fischer's exact test and P-values were adjusted applying Benjamini-Hochberg (P≤0.01) and Bonferroni (P≤0.05) multiple testing correction. (DOCX) [file pone.0112620.s002.docx]

**Table S2. Overrepresented GO functional categories in quiescent E2F2^-/-^ liver relative to WT controls.**

| Term | Count^a^ | %^b^ | P Value | Pop Hits | Pop Total | Fold Enrichment | Bonferroni | Benjamini | FDR |
| --- | --- | --- | --- | --- | --- | --- | --- | --- | --- |
| GO:0006810~transport | 445 | 15.34 | 0.0000 | 2325 | 13146 | 1.2512 | 0.0000 | 0.0000 | 0.0000 |
| GO:0006629~lipid metabolic process | 156 | 5.38 | 0.0000 | 692 | 13146 | 1.4737 | 0.0002 | 0.0001 | 0.0004 |
| GO:0044260~cellular macromolecule metabolic process | 777 | 26.79 | 0.0000 | 4471 | 13146 | 1.1361 | 0.0014 | 0.0005 | 0.0024 |
| GO:0009653~anatomical structure morphogenesis | 218 | 7.52 | 0.0000 | 1087 | 13146 | 1.3110 | 0.0081 | 0.0020 | 0.0139 |
| GO:0006082~organic acid metabolic process | 107 | 3.69 | 0.0000 | 474 | 13146 | 1.4757 | 0.0195 | 0.0039 | 0.0335 |
| GO:0042180~cellular ketone metabolic process | 109 | 3.76 | 0.0000 | 485 | 13146 | 1.4692 | 0.0198 | 0.0033 | 0.0340 |
| GO:0034641~cellular nitrogen compound metabolic process | 541 | 18.66 | 0.0000 | 3062 | 13146 | 1.1550 | 0.0242 | 0.0035 | 0.0417 |
| GO:0044249~cellular biosynthetic process | 516 | 17.79 | 0.0000 | 2917 | 13146 | 1.1564 | 0.0365 | 0.0046 | 0.0633 |
| GO:0006091~generation of precursor metabolites and energy | 65 | 2.24 | 0.0000 | 259 | 13146 | 1.6406 | 0.0401 | 0.0045 | 0.0698 |
| GO:0016042~lipid catabolic process | 39 | 1.34 | 0.0001 | 133 | 13146 | 1.9169 | 0.0568 | 0.0058 | 0.0995 |
| GO:0006793~phosphorus metabolic process | 173 | 5.97 | 0.0001 | 858 | 13146 | 1.3181 | 0.0574 | 0.0054 | 0.1006 |

^a^ Refers to the number of genes included in each overrepresented functional category. ^b^ Refers to the percentage of genes detected in each functional category relative to the total number of genes included in that particular category. Functional classification of deregulated genes was made using FatiGO+, a public domain web tool for finding significant associations of Gene Ontology (GO) terms within groups of genes. Statistical significance was determined by Fischer’s exact test and *P*-values were adjusted applying Benjamini-Hochberg (*P* ≤ 0.01) and Bonferroni (*P* ≤ 0.05) multiple testing correction.
