# Supplementary material for: The E2F2 Transcription Factor Sustains Hepatic Glycerophospholipid Homeostasis in Mice
Source: PLoS One. 2014 Nov 14;9(11):e112620. doi: 10.1371/journal.pone.0112620 (PMC4232400; doi:10.1371/journal.pone.0112620)
Supplement: Table S3 — E2F2 deletion deregulates the expression of a set of genes involved in lipid metabolism in quiescent liver. Genes (208) were extracted from the identified sequences listed in Table S1, categorized by our own criterion and shown in alphabetic order. Of them, 152 (73.1%) were up-regulated and 56 (26.9%) were down-regulated in the liver of E2F2 nullizygous mice in quiescence. (DOCX) [file pone.0112620.s003.docx]

**Table S3. E2F2 gene deletion deregulates the expression of a set of genes involved in lipid metabolism in quiescent liver.**

| **ID** | **Gene Title** | **Gene Symbol** | **Mean Ratio Log** |
| --- | --- | --- | --- |
|  |  |  |  |
|  | ***Fatty acid oxidation (mitochondrial and peroxisomal)*** |  |  |
| 1449047_at | 2-hydroxyacyl-CoA lyase 1 | Hacl1 | 0.411 |
| 1423858_a_at | 3-hydroxy-3-methylglutaryl-Coenzyme A synthase 2 | Hmgcs2 | 0.333 |
| 1453011_at | 3-hydroxybutyrate dehydrogenase, type 2 | Bdh2 | 0.378 |
| 1429339_a_at | acyl-Coenzyme A dehydrogenase family, member 10 | Acad10 | 0.433 |
| 1453206_at | acyl-Coenzyme A dehydrogenase family, member 9 | Acad9 | 0.467 |
| 1460216_at | acyl-Coenzyme A dehydrogenase, short chain | Acads | 0.433 |
| 1424184_at | acyl-Coenzyme A dehydrogenase, very long chain | Acadvl | 0.444 |
| 1416408_at | acyl-Coenzyme A oxidase 1, palmitoyl | Acox1 | 0.222 |
| 1420684_at | acyl-Coenzyme A oxidase 3, pristanoyl | Acox3 | 0.578 |
| 1423883_at | acyl-CoA synthetase long-chain family member 1 | Acsl1 | 0.289 |
| 1428082_at | acyl-CoA synthetase long-chain family member 5 | Acsl5 | 0.278 |
| 1437031_at | acyl-CoA synthetase long-chain family member 6 | Acsl6 | -1.078 |
| 1422479_at | acyl-CoA synthetase short-chain family member 2 | Acss2 | 0.433 |
| 1422651_at | adiponectin, C1Q and collagen domain containing | Adipoq | -1.611 |
| 1431989_at | acyloxyacyl hydrolase | Aoah | 0.578 |
| 1454661_at | ATP synthase, H+ transporting, mitochondrial F0 complex, subunit c (subunit 9), isoform 3 | Atp5g3 | 0.500 |
| 1423676_at | ATP synthase, H+ transporting, mitochondrial F0 complex, subunit d | Atp5h | 0.400 |
| 1449710_s_at | ATP synthase, H+ transporting, mitochondrial F1 complex, alpha subunit, isoform 1 | Atp5a1 | 0.211 |
| 1416567_s_at | ATP synthase, H+ transporting, mitochondrial F1 complex, epsilon subunit | Atp5e | 0.567 |
| 1416058_s_at | ATP synthase, H+ transporting, mitochondrial F1 complex, gamma polypeptide 1 | Atp5c1 | 0.411 |
| 1434934_at | ATP synthase mitochondrial F1 complex assembly factor 1 | Atpaf1 | 0.344 |
| 1439835_x_at | ATP-binding cassette, sub-family D (ALD), member 2 | Abcd2 | 1.089 |
| 1416679_at | ATP-binding cassette, sub-family D (ALD), member 3 | Abcd3 | 0.278 |
| 1460409_at | carnitine palmitoyltransferase 1a, liver | Cpt1a | 0.267 |
| 1416772_at | carnitine palmitoyltransferase 2 | Cpt2 | 0.344 |
| 1418321_at | dodecenoyl-Coenzyme A delta isomerase (3,2 trans-enoyl-Coenyme A isomerase) | Dci | 0.356 |
| 1448491_at | enoyl coenzyme A hydratase 1, peroxisomal | Ech1 | 0.311 |
| 1418862_at | enoyl Coenzyme A hydratase domain containing 3 | Echdc3 | 0.644 |
| 1448382_at | enoyl-Coenzyme A, hydratase/3-hydroxyacyl Coenzyme A dehydrogenase | Ehhadh | 0.467 |
| 1448499_a_at | epoxide hydrolase 2, cytoplasmic | Ephx2 | 0.311 |
| 1421234_at | HNF1 homeobox A | Hnf1a | 0.611 |
| 1460184_at | hydroxyacyl-Coenzyme A dehydrogenase | Hadh | 0.222 |
| 1452173_at | hydroxyacyl-Coenzyme A dehydrogenase/3-ketoacyl-Coenzyme A thiolase/enoyl-Coenzyme A hydratase (trifunctional protein), alpha subunit | Hadha | 0.333 |
| 1426522_at | hydroxyacyl-Coenzyme A dehydrogenase/3-ketoacyl-Coenzyme A thiolase/enoyl-Coenzyme A hydratase (trifunctional protein), beta subunit | Hadhb | 0.189 |
| 1451798_at | interleukin 1 receptor antagonist | Il1rn | -0.444 |
| 1449964_a_at | malonyl-CoA decarboxylase | Mlycd | 0.356 |
| 1417097_at | mitochondrial trans-2-enoyl-CoA reductase | Mecr | 0.289 |
| 1438399_at | peroxisomal biogenesis factor 5-like; peroxisomal membrane protein 3 | Pex5l | -1.322 |
| 1431012_a_at | peroxisomal delta3, delta2-enoyl-Coenzyme A isomerase | Peci | 0.300 |
| 1448910_at | peroxisomal trans-2-enoyl-CoA reductase | Pecr | 0.611 |
| 1455209_at | phosphoenolpyruvate carboxykinase 1, cytosolic | Pck1 | 0.644 |
| 1423109_s_at | solute carrier family 25 (mitochondrial carnitine/acylcarnitine translocase), member 20 | Slc25a20 | 0.233 |
| 1427483_at | solute carrier family 25 (mitochondrial carrier, phosphate carrier), member 24 | Slc25a24 | 1.700 |
| 1453133_at | solute carrier family 25 (mitochondrial carrier; adenine nucleotide translocator), member 31 | Slc25a31 | 1.589 |
| 1424316_at | solute carrier family 25 (mitochondrial thiamine pyrophosphate carrier), member 19 | Slc25a19 | 0.500 |
| 1416316_at | solute carrier family 27 (fatty acid transporter), member 2 | Slc27a2 | 0.433 |
| 1459740_s_at | uncoupling protein 2 (mitochondrial, proton carrier) | Ucp2 | 0.600 |
|  |  |  |  |
|  | ***Synthesis and precursor role of fatty acids*** |  |  |
| 1455395_at | 3-oxoacyl-ACP synthase, mitochondrial | Oxsm | 0.400 |
| 1429267_at | acyl-CoA thioesterase 11 | Acot11 | 0.611 |
| 1449457_at | acyl-CoA thioesterase 12 | Acot12 | 0.500 |
| 1423556_at | aldo-keto reductase family 1, member B7 | Akr1b7 | -0.611 |
| 1425771_at | aldo-keto reductase family 1, member D1 | Akr1d1 | 0.544 |
| 1422186_s_at | cytochrome b5 reductase 3 | Cyb5r3 | 0.178 |
| 1419094_at | cytochrome P450, family 2. subfamily c, polypeptide 37 | Cyp2c37 | 3.600 |
| 1455994_x_at | elongation of very long chain fatty acids (FEN1/Elo2, SUR4/Elo3, yeast)-like 1 | Elovl1 | 0.211 |
| 1437211_x_at | ELOVL family member 5, elongation of long chain fatty acids (yeast) | Elovl5 | 0.300 |
| 1441091_at | ELOVL family member 7, elongation of long chain fatty acids (yeast) | Elovl7 | -1.922 |
| 1434091_at | fatty acid amide hydrolase | Faah | 0.644 |
| 1417556_at | fatty acid binding protein 1, liver | Fabp1 | 0.467 |
| 1423680_at | fatty acid desaturase 1 | Fads1 | 0.222 |
| 1443838_x_at | fatty acid desaturase 2 | Fads2 | 0.533 |
| 1425216_at | free fatty acid receptor 2 | Ffar2 | -1.544 |
| 1452216_at | malonyl CoA:ACP acyltransferase (mitochondrial) | Mcat | 0.600 |
| 1422467_at | palmitoyl-protein thioesterase 1 | Ppt1 | 0.378 |
| 1417263_at | prostaglandin-endoperoxide synthase 2 | Ptgs2 | -1.322 |
| 1449112_at | solute carrier family 27 (fatty acid transporter), member 5 | Slc27a5 | 0.444 |
| 1415964_at | stearoyl-Coenzyme A desaturase 1 | Scd1 | 0.500 |
| 1444166_at | thyroid hormone responsive SPOT14 homolog (Rattus) | Thrsp | 0.478 |
| 1416352_s_at | trans-2,3-enoyl-CoA reductase | Tecr | 0.478 |
|  |  |  |  |
|  | ***Metabolism of glycerolipids*** |  |  |
| 1439259_x_at | abhydrolase domain containing 4 | Abhd4 | 0.344 |
| 1424451_at | acetyl-Coenzyme A acyltransferase 1B | Acaa1b | 0.589 |
| 1423598_at | ATPase, aminophospholipid transporter (APLT), class I, type 8A, member 1 | Atp8a1 | 1.656 |
| 1449145_a_at | caveolin 1, caveolae protein | Cav1 | 0.756 |
| 1452832_s_at | CDP-diacylglycerol synthase (phosphatidate cytidylyltransferase) 2 | Cds2 | 0.500 |
| 1442277_at | choline kinase alpha | Chka | 1.989 |
| 1435870_at | Choline phosphotransferase 1 | Chpt1 | -0.522 |
| 1455321_at | DDHD domain containing 1 | Ddhd1 | 0.489 |
| 1444320_at | DDHD domain containing 2 | Ddhd2 | 2.056 |
| 1455361_at | diacylglycerol kinase, beta | Dgkb | -0.767 |
| 1433564_at | diacylglycerol kinase, delta | Dgkd | 0.444 |
| 1443224_at | ectonucleotide pyrophosphatase/phosphodiesterase 2 | Enpp2 | 1.511 |
| 1436090_at | ectonucleotide pyrophosphatase/phosphodiesterase 6 | Enpp6 | -1.733 |
| 1437040_at | ethanolamine kinase 2 | Etnk2 | 0.289 |
| 1426370_at | fatty acyl CoA reductase 1 | Far1 | -2.278 |
| 1416694_at | glycerol kinase 2 | Gk2 | -1.789 |
| 1418444_a_at | glycerophosphodiester phosphodiesterase 1 | Gde1 | 0.644 |
| 1424077_at | glycerophosphodiester phosphodiesterase domain containing 1 | Gdpd1 | 0.511 |
| 1449526_a_at | glycerophosphodiester phosphodiesterase domain containing 3 | Gdpd3 | -1.678 |
| 1449440_at | lipin 3 | Lpin3 | 1.156 |
| 1434690_at | lysocardiolipin acyltransferase 1 | Lclat1 | 0.111 |
| 1423960_at | lysophosphatidylcholine acyltransferase 3 | Lpcat3 | 0.411 |
| 1450872_s_at | lysosomal acid lipase A | Lipa | 0.344 |
| 1438386_x_at | methionine adenosyltransferase II, alpha | Mat2a | 0.367 |
| 1448196_at | methionine adenosyltransferase II, beta | Mat2b | 0.500 |
| 1426785_s_at | monoglyceride lipase | Mgll | 0.356 |
| 1457313_at | oculocerebrorenal syndrome of Lowe | Ocrl | 1.700 |
| 1459217_at | oncostatin M receptor | Osmr | -0.878 |
| 1427379_at | patatin-like phospholipase domain containing 6 | Pnpla6 | 0.611 |
| 1457355_at | patatin-like phospholipase domain containing 8 | Pnpla8 | 0.778 |
| 1448318_at | perilipin 2 | Plin2 | 0.333 |
| 1416424_at | perilipin 3 | Plin3 | 0.311 |
| 1420493_a_at | phosphate cytidylyltransferase 2, ethanolamine | Pcyt2 | 0.411 |
| 1428154_s_at | phosphatidic acid phosphatase type 2 domain containing 1B | Ppapdc1b | 0.611 |
| 1422619_at | phosphatidic acid phosphatase type 2A | Ppap2a | -0.256 |
| 1429514_at | phosphatidic acid phosphatase type 2B | Ppap2b | 0.400 |
| 1420984_at | phosphatidylcholine transfer protein | Pctp | 0.578 |
| 1421023_at | phosphatidylinositol 3-kinase, C2 domain containing, alpha polypeptide | Pik3c2a | -0.356 |
| 1421704_a_at | phosphatidylinositol 3-kinase, C2 domain containing, gamma polypeptide | Pik3c2g | -1.189 |
| 1433462_a_at | phosphatidylinositol 4-kinase type 2 alpha | Pi4k2a | 0.411 |
| 1439197_at | phosphatidylinositol 4-kinase, catalytic, beta polypeptide | Pi4kb | -1.278 |
| 1437999_x_at | phosphatidylinositol glycan anchor biosynthesis, class Q | Pigq | 0.867 |
| 1447382_at | phosphatidylinositol glycan anchor biosynthesis, class T | Pigt | -0.889 |
| 1427466_at | phosphatidylinositol glycan anchor biosynthesis, class U | Pigu | -1.044 |
| 1452940_x_at | phosphatidylinositol transfer protein, cytoplasmic 1 | Pitpnc1 | -0.556 |
| 1437724_x_at | phosphatidylinositol transfer protein, membrane-associated 1 | Pitpnm1 | -0.333 |
| 1435462_at | phosphatidylinositol-specific phospholipase C, X domain containing 2 | Plcxd2 | -0.278 |
| 1449739_at | phosphatidylserine synthase 1 | Ptdss1 | -1.400 |
| 1434586_a_at | phosphatidylserine synthase 2 | Ptdss2 | 0.233 |
| 1444647_at | phospholipase A2, activating protein | Plaa | 1.767 |
| 1421325_at | phospholipase A2, group IIF | Pla2g2f | -0.600 |
| 1456047_at | phospholipase A2, group IVB (cytosolic); jumonji domain containing 7 | Pla2g4b | 0.633 |
| 1429862_at | phospholipase A2, group IVE | Pla2g4e | 0.533 |
| 1455408_at | phospholipase A2, group IVF | Pla2g4f | -0.900 |
| 1436335_at | phospholipase C, eta 2 | Plch2 | -0.578 |
| 1432405_a_at | phospholipase C, zeta 1 | Plcz1 | -2.056 |
| 1416013_at | phospholipase D family, member 3 | Pld3 | 0.344 |
| 1417963_at | phospholipid transfer protein | Pltp | 0.611 |
| 1448489_at | platelet-activating factor acetylhydrolase 2 | Pafah2 | -0.233 |
| 1442878_at | similar to Peroxiredoxin-6 (Antioxidant protein 2) (1-Cys peroxiredoxin) | Prdx6 | -0.444 |
| 1437037_x_at | staphylococcal nuclease and tudor domain containing 1 | Snd1 | -0.456 |
|  |  |  |  |
|  | ***Metabolism of sphingolipids*** |  |  |
| 1440331_at | 3-ketodihydrosphingosine reductase | Kdsr | 1.278 |
| 1449847_a_at | collagen, type IV, alpha 3 (Goodpasture antigen) binding protein | Col4a3bp | -1.122 |
| 1452907_at | galactosylceramidase | Galc | 0.411 |
| 1418050_at | glycosylphosphatidylinositol specific phospholipase D1 | Gpld1 | 0.189 |
| 1433495_at | glycosyltransferase 25 domain containing 1 | Glt25d1 | 0.789 |
| 1460180_at | hexosaminidase B | Hexb | 0.311 |
| 1430819_at | N-acylethanolamine acid amidase | Naaa | 0.833 |
| 1448506_at | serine (or cysteine) peptidase inhibitor, clade A, member 6 | Serpina6 | -0.333 |
| 1446310_at | serine palmitoyltransferase, long chain base subunit 3 | Sptlc3 | -1.033 |
| 1438665_at | sphingomyelin phosphodiesterase 3, neutral | Smpd3 | 0.400 |
| 1436499_at | sphingomyelin synthase 1 | Sgms1 | -0.400 |
| 1449198_a_at | ST3 beta-galactoside alpha-2,3-sialyltransferase 5 | St3gal5 | 0.533 |
| 1454246_at | ST3 beta-galactoside alpha-2,3-sialyltransferase 6 | St3gal6 | 1.100 |
| 1449468_at | ST6 (alpha-N-acetyl-neuraminyl-2,3-beta-galactosyl-1,3)-N-acetylgalactosaminide alpha-2,6-sialyltransferase 5 | St6galnac5 | -1.322 |
| 1423115_at | ST6 (alpha-N-acetyl-neuraminyl-2,3-beta-galactosyl-1,3)-N-acetylgalactosaminide alpha-2,6-sialyltransferase 6 | St6galnac6 | 0.311 |
| 1420447_at | sulfotransferase family 1E, member 1 | Sult1e1 | -1.778 |
| 1419138_at | UDP-Gal:betaGlcNAc beta 1,3-galactosyltransferase, polypeptide 4 | B3galt4 | 1.111 |
| 1428397_at | UDP-Gal:betaGlcNAc beta 1,3-galactosyltransferase, polypeptide 5 | B3galt5 | 1.400 |
| 1439863_at | UDP-glucose ceramide glucosyltransferase | Ugcg | -1.522 |
|  |  |  |  |
|  | ***Steroid metabolism and bile secretion*** |  |  |
| 1426635_at | acyl-Coenzyme A binding domain containing 3 | Acbd3 | -0.222 |
| 1417208_at | alpha-methylacyl-CoA racemase | Amacr | 1.478 |
| 1449818_at | ATP-binding cassette, sub-family B (MDR/TAP), member 4 | Abcb4 | 0.411 |
| 1452233_at | ATP-binding cassette, sub-family C (CFTR/MRP), member 1 | Abcc1 | -0.900 |
| 1450109_s_at | ATP-binding cassette, sub-family C (CFTR/MRP), member 2 | Abcc2 | 1.456 |
| 1449081_at | carboxylesterase 3 | Ces3 | 0.556 |
| 1459865_x_at | carboxylesterase 7 | Ces7 | 1.022 |
| 1457984_at | corticotropin releasing hormone | Crh | 1.556 |
| 1416613_at | cytochrome P450, family 1, subfamily b, polypeptide 1 | Cyp1b1 | 0.656 |
| 1439947_at | cytochrome P450, family 11, subfamily a, polypeptide 1 | Cyp11a1 | -1.133 |
| 1417017_at | cytochrome P450, family 17, subfamily a, polypeptide 1 | Cyp17a1 | 0.422 |
| 1449920_at | cytochrome P450, family 19, subfamily a, polypeptide 1 | Cyp19a1 | -0.756 |
| 1444138_at | cytochrome P450, family 2, subfamily r, polypeptide 1 | Cyp2r1 | -0.467 |
| 1418866_at | cytochrome P450, family 24, subfamily a, polypeptide 1 | Cyp24a1 | -0.544 |
| 1427372_at | cytochrome P450, family 27, subfamily b, polypeptide 1 | Cyp27b1 | 1.256 |
| 1421075_s_at | cytochrome P450, family 7, subfamily b, polypeptide 1 | Cyp7b1 | -0.533 |
| 1451559_a_at | dehydrogenase/reductase (SDR family) member 4 | Dhrs4 | 0.211 |
| 1417298_at | emopamil binding protein-like | Ebpl | 0.367 |
| 1434642_at | hydroxysteroid (17-beta) dehydrogenase 11 | Hsd17b11 | 0.167 |
| 1417369_at | hydroxysteroid (17-beta) dehydrogenase 4 | Hsd17b4 | 0.356 |
| 1449038_at | hydroxysteroid 11-beta dehydrogenase 1 | Hsd11b1 | 0.500 |
| 1451122_at | isopentenyl-diphosphate delta isomerase | Idi1 | -0.233 |
| 1441824_at | membrane bound O-acyltransferase domain containing 1 | Mboat1 | -0.700 |
| 1436883_at | membrane-bound transcription factor peptidase, site 2 | Mbtps2 | 0.478 |
| 1460246_at | methyl CpG binding protein 2 | Mecp2 | 0.311 |
| 1451927_a_at | mitogen-activated protein kinase 14 | Mapk14 | -0.889 |
| 1420410_at | nuclear receptor subfamily 5, group A, member 2 | Nr5a2 | -0.611 |
| 1426968_a_at | retinol dehydrogenase 10 (all-trans) | Rdh10 | 0.167 |
| 1420541_at | retinol dehydrogenase 16 | Rdh16 | 0.456 |
| 1427963_s_at | retinol dehydrogenase 9 | Rdh9 | -0.311 |
| 1424716_at | retinol saturase (all trans retinol 13,14 reductase) | Retsat | 0.522 |
| 1450261_a_at | solute carrier family 10 (sodium/bile acid cotransporter family), member 1 | Slc10a1 | 0.322 |
| 1436302_at | solute carrier family 10 (sodium/bile acid cotransporter family), member 7 | Slc10a7 | 0.767 |
| 1429239_a_at | StAR-related lipid transfer (START) domain containing 4 | Stard4 | -0.400 |
| 1454649_at | steroid 5 alpha-reductase 1 | Srd5a1 | 0.356 |
| 1445180_at | steroid 5 alpha-reductase 3 | Srd5a3 | 1.133 |
| 1426219_at | sterol carrier protein 2, liver | Scp2 | 0.533 |
| 1417697_at | sterol O-acyltransferase 1 | Soat1 | 0.956 |
| 1426744_at | sterol regulatory element binding factor 2 | Srebf2 | 0.556 |
| 1451457_at | sterol-C5-desaturase (fungal ERG3, delta-5-desaturase) homolog | Sc5d | 0.411 |
|  |  |  |  |
|  | ***Plasma lipid transport and metabolism*** |  |  |
| 1436879_x_at | alpha fetoprotein | Afp | -2.133 |
| 1450085_at | angiopoietin-like 2 | Angptl2 | -0.611 |
| 1424485_at | angiopoietin-like 3 | Angptl3 | 0.600 |
| 1417610_at | apolipoprotein A-V | Apoa5 | 0.211 |
| 1455593_at | apolipoprotein B | Apob | 0.489 |
| 1417889_at | apolipoprotein B mRNA editing enzyme, catalytic polypeptide 2 | Apobec2 | 1.267 |
| 1418278_at | apolipoprotein C-III | Apoc3 | 0.467 |
| 1452800_a_at | apolipoprotein O | Apoo | 0.522 |
| 1449302_at | ATP-binding cassette, sub-family A (ABC1), member 2 | Abca2 | 1.422 |
| 1440879_at | ATP-binding cassette, sub-family A (ABC1), member 9 | Abca9 | 0.800 |
| 1423166_at | CD36 antigen | Cd36 | 0.378 |
| 1419806_at | high density lipoprotein (HDL) binding protein | Hdlbp | -0.722 |
| 1448655_at | low density lipoprotein receptor-related protein 1 | Lrp1 | 0.622 |
| 1416836_at | low-density lipoprotein receptor-related protein 10 | Lrp10 | 0.611 |
| 1436083_at | low density lipoprotein receptor-related protein 3 | Lrp3 | 1.067 |
| 1426288_at | low density lipoprotein receptor-related protein 4 | Lrp4 | 0.478 |
| 1439821_at | Lrp2 binding protein | Lrp2bp | 0.789 |
| 1454704_at | scavenger receptor class B, member 2 | Scarb2 | 0.333 |
| 1426258_at | similar to sortilin-related receptor, LDLR class A repeats-containing | Sorl1 | -0.656 |
| 1447602_x_at | sulfatase 2 | Sulf2 | 1.456 |

Genes (208) were extracted from the identified sequences listed in Table S1, categorized by our own criterion and shown in alphabetic order. Of them, 152 (73.1%) were up-regulated and 56 (26.9%) were down-regulated in the liver of E2F2 nullizygous mice in quiescence.
